# Monitoring Anti-NS1 Antibodies in West Nile Virus-Infected and Vaccinated Horses

**DOI:** 10.1155/2018/8309816

**Published:** 2018-09-25

**Authors:** Belén Rebollo, Javier Sarraseca, Sylvie Lecollinet, Nabil Abouchoaib, Javier Alonso, Ignacio García-Bocanegra, Antonio J. Sanz, Ángel Venteo, Miguel A. Jiménez-Clavero

**Affiliations:** ^1^INGENASA (Inmunología y Genética Aplicada, SA), Madrid 28037, Spain; ^2^ANSES, Animal Health Laboratory, Maisons-Alfort 94706, France; ^3^Laboratoire Regional d'Analyses et de Recherches de Casablanca, ONSSA, Casablanca, Morocco; ^4^Faculty of Sciences, University Mohammed V, Rabat, Morocco; ^5^Laboratorio de Producción y Sanidad Animal de Jerez de la Frontera (Cádiz), Spain; ^6^Facultad de Veterinaria, Universidad de Córdoba, Spain; ^7^Centro de Investigación en Sanidad Animal, Instituto Nacional de Investigación y Tecnología Agraria y Alimentaria (INIA-CISA), Valdeolmos 28130, Spain; ^8^CIBER Epidemiología y Salud Pública (CIBERESP), Spain

## Abstract

West Nile virus (WNV) is a zoonotic arboviral pathogen affecting humans, birds, and horses. Vaccines are available for veterinary use, which efficiently prevent the infection in horses. Most common diagnostic tools rely on the identification of the agent (RT-PCR, virus isolation), or on the detection of antibodies (IgM and IgG) recognizing structural proteins of the virus or neutralizing virus infection in cell cultures (virus-neutralization tests). The recent emergence of WNV in different parts of the world has resulted in an increase in the vaccination of horses in many countries. Methods for differentiation between infected and vaccinated animals (“DIVA” assays) would be useful for surveillance and control purposes but are still not available. A usual approach in this regard is the use of antibodies to nonstructural proteins as markers of nonvaccinated, infected animals, and the nonstructural NS1 protein of WNV has been proposed as a candidate for such a marker. The aim of this study was to test the hypothesis that NS1 can be a useful antigen in DIVA assays for differentiating WNV vaccinated and infected horses in field conditions. For that, we examined serum samples from either vaccinated and infected horses both from experimental infections/vaccinations (under controlled conditions) and from the field, exposed to natural infection or vaccinated in response to a risk of infection. The overall conclusion of the study is that NS1 antigen can effectively differentiate WNV infected from vaccinated horses in experimental (controlled) conditions, but this differentiation might be difficult depending on the conditions prevailing in the field.

## 1. Introduction

West Nile virus (WNV) is a mosquito-borne zoonotic arbovirus belonging to the genus* Flavivirus* in the family Flaviviridae. In nature, WNV is maintained in a rural sylvatic cycle between mosquitoes (mainly* Culex*) and wild birds. Horses and humans can be incidentally affected but are considered dead-end hosts because in these species the viremia levels are not enough to infect mosquitoes when taking a blood meal.

WNV genome is a single-stranded (ss) RNA molecule of positive polarity that encodes for 3 structural proteins (C, M, E; capsid, membrane, and envelope proteins, respectively) and 7 nonstructural (NS) proteins (NS1, NS2a, NS2b, NS3, NS4a, NS4b, and NS5). The host humoral immune response is directed not only to the structural proteins but also to the nonstructural ones. During the infection the antibodies produced are mainly targeted against E, NS1, and NS3 proteins [1].

The virus can cause a severe neuroinvasive disease in approximately 8% of exposed horses [2]. The surveillance programs usually rely mostly on serological monitoring of horses, mainly in endemic areas where horses can be previously infected or vaccinated. The evidence of IgM antibodies or seroconversion (indicated by a 4-fold increase in antibody titres in 2 serial samples from the same individual obtained 2-3 weeks apart) is necessary to confirm WNV infection in horses. However, due to logistic constrains, it is not always possible to obtain paired sera for surveillance purposes.

In Europe, where the virus has reemerged and is active in many countries, there are three different vaccines licensed that prevent WNV infection in horses: a chemically inactivated virus (Duvaxyn® WNV, now Equip® WNV, Zoetis, Louvain-la-Neuve, Belgium) a live recombinant canarypox virus vaccine that expresses the genes encoding for the structural preM/E proteins of WNV (Proteq® West Nile, Merial, Lyon, France), and an inactivated chimeric WNV-YF vaccine, where the yellow fever virus vaccine strain YF-17D presents the genes for the structural proteins E and prM of WNV (Equilis® West Nile, Intervet, Boxmeer, The Netherlands). In Morocco, where active circulation of WNV is also observed, an additional inactivated WNV vaccine (WNVVac®, based on Maroc 96-107 strain, Biopharma, Rabat, Morocco) is licensed, along with Recombitek® Equine West Nile, which is essentially identical to Proteq® West Nile (both are manufactured by Merial) but the first is supplied freeze-dried and requires resuspension prior to administration whereas the second is supplied as liquid suspension ready to use. All these vaccines elicit an immune response characterized by antibodies that efficiently recognize the WNV structural proteins, which constitute the immunogenic part of the vaccine formulation. [3–5]. Furthermore, these vaccines are not expected to raise antibodies to the nonstructural (NS) proteins. Hence, methods for differentiation of WNV-infected from vaccinated horses (“DIVA” methods) which are still not available could be developed, at least hypothetically, based on measuring the differential reactivity of horse serum antibodies to E and NS proteins. These methods could allow monitoring of virus circulation in horse populations in regions where vaccination is in place. In this regard, NS1 protein of WNV has been proposed as a DIVA marker for WNV infection, and preliminary evidence has been obtained in experimentally infected/vaccinated rabbits and chickens, supporting this assumption [6]. Another recent study using a NS1-based protein microarray did not find relevant differences in serum antibody titres to NS1 between horses experimentally infected and vaccinated with inactivated virus vaccine [7]. Apart from this, to the best of our knowledge, no other study to date has focused on differential serological responses to WNV vaccines with respect to WNV infections in horses, despite the many gaps still remaining on this issue, e.g., absence of data from field sera or from different types of vaccines. The purpose of the present study was to test whether NS1-specific antibodies can be used, alone or in combination with other serological tools, to effectively differentiate WNV-infected and vaccinated horses by means of their serological profiles, including NS1 antibodies, using easy and affordable serological techniques (ELISA). For that, we have developed an ELISA test for detection of antibodies to NS1 in horse sera and compared the reactivity of a collection of serum samples from vaccinated, infected, and control (noninfected, nonvaccinated) horses, against NS1 and the structural proteins. This collection of samples included both experimental and field samples from diverse origins and different WNV vaccines, either inactivated (Equip®, WNVvac®) or recombinant (Proteq®, Recombitek®).

## 2. Materials and Methods

### 2.1. Expression and Purification of Recombinant Proteins

Two recombinant baculovirus clones were generated to express, respectively, the NS1 protein and the structural proteins in the form of “virus-like particles” (VLP) of WNV, as previously described [8, 9]. Briefly the NS1-coding region from NY99 034EDV “crow” strain was cloned into the baculovirus expression vector pAcHLA, expressed in Sf9 insect cells and purified by Ni-affinity column. The C and prM-E protein-coding genes from the same WNV strain were cloned in the multiple expression vector pBac4x-1, expressed in Sf9 cells and purified by ultracentrifugation in a sucrose cushion.

### 2.2. Serum Samples

Two different panels of samples were used in this study, one from horses infected or vaccinated under experimental premises (“experimental sera”) and another one collected in the field from either naturally infected or vaccinated horses (“field sera”) (details in Table 1).

The panel of experimental sera consisted of two different groups.

*Group A1.* These samples, provided by the European Reference Laboratory for equine diseases, ANSES, Maisons-Alfort, France, were obtained from two horses experimentally inoculated with an infectious dose of WNV Lineage 1 (L1 Israel ′98 strain) and L2 (Austria′08 strain), respectively, and bled at 0, 8, 11, 14, 20, 35, and 58 days after infection, as described elsewhere [10].

*Group B1.* These serum samples were obtained from five horses, each vaccinated with 2 doses of Duvaxyn® WNV, Pfizer (now Equip® WNV, Zoetis) following manufacturer's recommendations. Briefly, five asymptomatic horses from a sentinel group, linked to a surveillance program held by the animal health authority in Andalusia, Spain (Consejería de Agricultura y Pesca, Junta de Andalucía), and that were previously confirmed as negative for WNV antibodies by INgezim West Nile Compac, were vaccinated with 2 doses of the inactivated vaccine following the manufacturer instructions and bled at 0, 14, 21, 28, 42, 56, 70, 85, and 115 days after vaccination.

The panel of field serum samples corresponded to either naturally infected horses from different countries of known WNV circulation (A2: France, n=22; A3: Italy, n=17; A4: Morocco, n=24 and A5: Spain: n=52) or horses in different locations in Spain and Morocco (n=163) that were vaccinated to prevent WNV infection, due to the risk posed by WNV circulation. These horses were vaccinated with four types of vaccines: Duvaxyn® WNV, Proteq® West Nile, Recombitek® Equine West Nile, and WNV Vac in different combinations and schedules as detailed in Table 1. Briefly, 26 serum samples were obtained from horses vaccinated with inactivated Duvaxyn WNV vaccine (group B2); 30 serum samples were from horses vaccinated with Proteq West Nile vaccine (2 doses, 3 weeks apart, group B3); 75 serum samples were from horses vaccinated with Proteq West Nile as in group B3 but receiving a recall vaccination one year later (that is, 2 complete vaccination schedules with Proteq West Nile vaccine in 2 consecutive years, group B4); 13 samples were from horses vaccinated with WNVVac inactivated vaccine (2 doses, 3 weeks apart) (group B5), and 19 samples were from horses vaccinated with Recombitek West Nile (2 doses, 3 weeks apart) with a recall vaccination (second year) with WNVVac inactivated WNV Maroc 96-107 vaccine.

Samples from the infected horses were collected as part of the surveillance programs for West Nile fever in the mentioned countries. Their infection status was determined by means symptoms and serology (IgM and/or total antibodies to WNV) [11].

*Group C (Control Group)*. Sera from noninfected, nonvaccinated horses (n=96) with no antibodies to WNV, confirmed by WNV competitive and IgM ELISA assays are described below.

### 2.3. Indirect ELISA Tests for Detection of Anti-NS1 and Antistructural Protein (VLP) Antibodies

ELISA plates (High Binding microtiter plates, Costar Stripwell™ Flat Bottom) were coated with purified recombinant NS1 or VLPs at 0.3 *μ*g protein/well, adsorbed overnight at 4°C in carbonate-bicarbonate buffer, pH 9.6. Then, after a washing step wells were blocked with 5% BSA in PBS for 1 h at 25°C. The plates were then incubated during 30 min with the serum samples at 1/200 dilution in a PBS-Tween 20 plus 1% BSA. After a washing step to remove the unbound material, a horseradish peroxidase- (HRPO-) conjugated anti-horse IgG monoclonal antibody (1DA6, INGENASA, Madrid, Spain) was added and allowed to react for 30 min. After a further washing step, TMB (3,3′,5,5′-Tetramethylbenzidine) substrate solution was added to each well. Colour was allowed to develop for 10 minutes, and then the reaction was stopped by adding 1N H_2_SO_4_ and the plate was read at 450 nm wavelength.

The indirect ELISAs for NS1- and VLP-specific antibody detection were set up with the experimental sera (groups A1 and B1). The amount of coated protein and the diluents were optimized to allow the best difference between infected and vaccinated and the lowest background with the negatives. Optical densities at 450 nm (ODs) above 0.5 units were considered positive in both indirect ELISAs.

### 2.4. Competitive and IgM ELISA Assays

For overall detection of antibodies to WNV, horse serum samples were analysed by a commercial blocking ELISA (INgezim® WNV Compac, INGENASA, Madrid, Spain). For anti-WNV-specific IgM antibody detection, sera were subjected to analysis by IgM-capture ELISA (INgezim® WNV IgM, INGENASA, Madrid, Spain) following manufacturer's instructions.

### 2.5. Data Analysis and Statistics

For each individual serum sample, OD ratios were calculated by dividing the net OD (resulting from background (obtained with negative sera) OD substraction of each individual OD value) obtained from the VLP ELISA by the net OD value from the NS1 ELISA. A cut-off ratio value of 4 was set to allow for a better discrimination between infected/vaccinated groups.

Statistical comparisons were done using the Mann-Whitney* U* test for nonparametric data of continuous variables (OD) and Fisher exact test for discontinuous variables (distribution of sera by VLP/NS1 OD ratio R).

## 3. Results

### 3.1. Serological Results under Experimental Conditions

**Figure 1:**
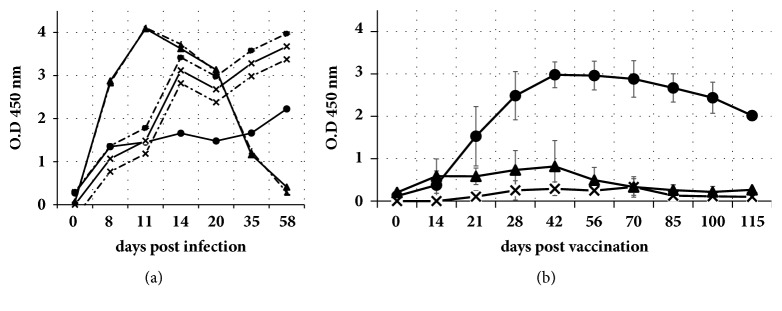
Results of serological examination of serum series from A1 and B1 groups: (a) two horses experimentally infected with either Israel ′98 L1 WNV strain (dashed lines) or Austria′08 L2 WNV strain (continuous lines) and (b) five horses experimentally inoculated with Duvaxyn inactivated vaccine, as described in Materials and Methods section. Different curves correspond to optical density results (ordinate scale) obtained by IgM-capture ELISA (triangles), indirect IgG-VLP ELISA (circles), and indirect IgG-NS1 ELISA (crossed dots). In (b) each dot represents mean (bars: standard deviation) of five determinations, one for each horse.

The horses from group A1 were subjected to experimental inoculation of WNV (either lineage 1 or lineage 2) seroconverted at 8 days after infection (dpi), ascertained by either competitive ELISA (not shown) or IgM-capture ELISA. IgM antibodies peaked at 11 dpi. Indirect ELISAs, performed in parallel, revealed NS1-specific and anti-VLP IgG antibodies also at 8 dpi. Both NS1 and VLP antibodies rose fast up to 14 dpi and then rose slowly up to the end of the experiment (58 dpi). However, IgM antibodies decayed after 35 days upon infection (Figure 1(a)).

The horses from group B1, vaccinated under experimental conditions with Duvaxyn® WNV vaccine, showed seroconversion to WNV structural proteins, ascertained by either competitive ELISA (14-21 days after vaccination, dpv) (not shown), IgM-capture ELISA (14-21 dpv), or indirect ELISA for VLP-specific IgG antibodies (21 dpv). However, they did not show any detectable IgG reactivity to NS1 at any time after vaccination (Figure 1(b)).

**Figure 2:**
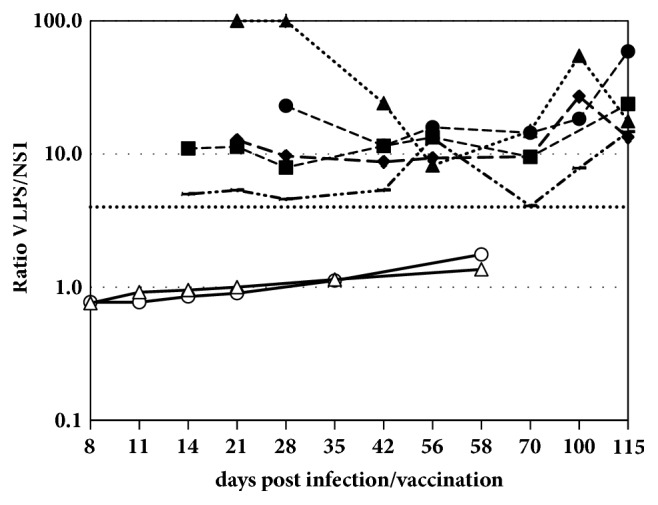
Comparison of serological results, expressed as VLP/NS1 OD ratios (net OD in VLP ELISA divided by net OD in NS1 ELISA) obtained with different series of sera from five horses experimentally inoculated with inactivated WNV vaccine (dashed lines) and two experimentally infected horses (continuous lines), as described in the Materials and Methods section. The horizontal line set at a ratio (R)=4 depicts the cut-off value selected in this study for differentiation of vaccinated and infected horses.

VLP/NS1 OD ratios (R), calculated for each individual sample, were compared in order to verify if the serological profiles may reflect the infection versus vaccination status of the sampled horse. All R values obtained from WNV-seropositive sera from the infected horses (i.e., those giving positive result in the WNV competition ELISA, INgezim® WNV Compac) were lower than 4, whereas sera from horses experimentally vaccinated yielded R values above 4 in all horses (Figure 2).

### 3.2. Serological Results in Field Conditions

All horses naturally infected with WNV (groups A2 to A5, n=115) showed positive results in WNV competition ELISA and most of them (n=110, 94%) were also positive by IgM-capture ELISA and were also strongly positive for IgG antibodies to WNV-VLPs. However, only 77 (66.9%) reacted in the ELISA for NS1 IgG antibodies (Table 2). No significant differences were found in the serological results observed between horses from different country of origin.

When sera from field vaccinated horses (groups B2 to B6) were analysed for anti-NS1 antibodies, as revealed by the indirect NS1 ELISA, no statistically significant difference was observed related to vaccine type or schedule: the NS1 antibody signals were low in general, with a mean OD=0.31 units and 22 positives out of 164 sera analysed (13.4%) (Table 2). Statistically significant differences (p<0.05) were found between field-infected and vaccinated horses with regard to NS1 ELISA ODs (infected: 0.72, n=115; vaccinated: 0.31, n=163)

When these groups were analysed for anti-VLP antibodies, as revealed by the indirect VLP ELISA, overall, higher OD signals (means ranging from 1.1 to 2.5 units) were observed in all groups except group B3, which showed a mean OD of 0.72 units, the lowest amongst the groups of vaccinated horses (Table 2), although the differences observed between all these groups were not significant statistically. Also, no statistically significant difference was observed between field-infected and vaccinated horses with regard to VLP antibody signals.

Then VLP/NS1 ELISA OD (R) ratios were compared between these groups of samples. Overall, as expected, sera from naturally infected horses showed lower R than sera from horses receiving vaccination in the field. However, in contrast with the results observed in experimentally infected/vaccinated horses (group A1 versus group B1), the field groups were not homogeneous with regard to this parameter. While 80% of the field sera from WNV-infected horses (92 out of 115 sera) exhibited R< 4, in field vaccinated horse the 62.6% (61 out of 163 sera) of samples showed R> 4 in field vaccinated horses (Table 2). This overall difference between field-infected and vaccinated horses was statistically significant (p<0.05). However, no significant difference was found amongst the infected groups (A2-A5) by country of origin. With regard to the field vaccinated groups, these exhibited some differences on R: groups receiving inactivated vaccines alone; that is, groups B2 and B6, showed higher percentages of samples displaying R<4 (50% and 30.7%, respectively) than those receiving recombinant live vaccines with recall vaccinations with either the same formulation as in primovaccination (group B4) or a different, inactivated vaccine (group B5), with lower (18.6%) or null percentages, respectively, of samples with R< 4. Finally, one of the groups of horses vaccinated in the field, B3, receiving a single vaccination schedule with a live recombinant vaccine, displayed an unexpected 100% of sera with R< 4.

As for the control group (group C, noninfected, nonvaccinated horses, n=96), they showed negative results in all the serological tests employed.

## 4. Discussion

This study describes the results obtained with different serological analyses for WNV-specific antibodies, including results on NS1 antibody recognition, over a wide panel of horse samples. The objective was to assess if differences can be found between WNV-infected and vaccinated animals on the basis of the presence and levels of NS1 antibodies as measured by the specific NS1-ELISA developed to this aim, either alone or in combination with other serological tests. Differential serological tests leading to a DIVA (“differentiation between infected and vaccinated assays”) strategy, as has been established for other infectious agents such as foot-and-mouth disease virus [12], would be highly valuable not only for enabling a more accurate diagnosis of WNV disease, allowing differentiating genuine infections from vaccinations, but also in disease surveillance for monitoring virus circulation in horse populations in regions where vaccination is in place. In principle, vaccinated horses are not expected to develop anti-NS1 antibodies, as the vaccines are based on either inactivated virus preparations constituting mainly of virions composed by structural proteins, or recombinant vaccines made out by viral vectors in which only structural proteins from WNV have been incorporated, and thus lacking WNV NS proteins. However, in vaccines based on inactivated virus, trace amounts of NS1 in the vaccine preparations may cause a rise in NS1-specific antibodies even in vaccinated animals. In a recent study, Cleton and cols found NS1-specific antibodies in horses vaccinated with whole inactivated virus (Duvaxyn® WNV) vaccine [7]. These authors hypothesized that trace amounts of NS1 in the vaccine preparation might be responsible for the antibodies found. If this hypothesis is confirmed, then NS1-based DIVA strategies may be hampered, at least for this type of vaccines. However, it may occur that different vaccine batches may contain different trace amounts of NS1, some resulting immunogenic to elicit an anti-NS1 antibody response, some not, which consequently may lead to different serological outcomes. Furthermore, there are other vaccine formats, particularly recombinant ones, which may not be affected by this problem. It was therefore worthwhile to analyse more vaccination formulations, such as those examined in this study, in order to further assess if NS1 antibody response is a useful parameter to substantiate serological tools enabling DIVA methods that may enrich the West Nile fever diagnostic toolkit.

To evaluate the usefulness of NS1 antibodies to develop a DIVA strategy in WNV disease diagnosis, an approach integrating not only experimental but also field sera is mandatory. The samples examined in this study included experimental as well as field settings. In the first instance, the vaccine used was an inactivated virus vaccine, Duvaxyn®. In order to measure NS1 specific antibodies, an indirect NS1-ELISA was developed. Moreover, to allow for a better comparison of the data, a similar ELISA was developed to assess antibody levels to WNV structural proteins (“virus-like particles”, VLPs) in parallel with NS1 ones. First it was shown that the antibody response to NS1 was much lower in the vaccinated than in the infected horses, while the antibodies to VLPs were comparably high in both groups. This result clearly indicated that the inactivated virus vaccine was not efficient in eliciting NS1 antibodies in horses. At the same time, it showed that the vaccine elicited a good immunity to the structural proteins, comparable to the response against the infection. These results indicated that a DIVA method based on measuring anti-NS1 antibody responses could be feasible, at least with this type of vaccine. Such a different outcome from the results published by Cleton et al. referred above may perhaps be explained by different trace amounts of NS1 in the different vaccine batches used in each experiment.

Furthermore, these results suggested that the ratio between the ODs obtained from both ELISAs may better substantiate differences between the vaccination versus infection status of the horses. Therefore, we calculated the VLP/NS1 OD ratios (R) by dividing the OD obtained in the VLP ELISA by the OD obtained in the NS1 ELISA, and the results were depicted in a graph (Figure 2) which clearly indicated that sera from vaccinated horses reached R values always higher than 4 and in sera from infected horses the R values observed were lower than 4. Hence, an R= 4 was selected as cut-off for further studies.

In the experimentally infected horses, the indirect ELISAs developed for this study detected horse IgG molecules reacting specifically with VLP and NS1 antigens, respectively, starting from 8 days after infection to the end of the experiment (58 dpi). The IgM antibody response to WNV measured in parallel was first detected also at 8 dpi, peaked at 11 dpi, and declined slowly thereafter, to yield a faint signal at 58 dpi. This kinetic is in agreement with similar experiments already published, which pointed out that specific IgM levels in horses began to rise not earlier than 7 days after infection and continued to increase up to 13 dpi [13]. In comparison to IgM kinetics, IgG responses are known to be slightly delayed during the infection. However, as judged by the data obtained, in this case this delay is so low that would likely have little effect on the sensitivity of the indirect ELISAs, as most IgM-positive sera would be expected to give IgG positive signals in indirect ELISA also. This point is relevant since most cases of WNV disease in horses are confirmed in the laboratory based on an IgM-positive ELISA result, so the majority of sera requiring DIVA analysis would be expected to be IgM-positive.

In contrast with the results obtained in the experimental samples, in field samples the results were not homogeneous with regard to NS1 ELISA; i.e., not all samples from infected horses were positive and not all samples from vaccinated horses were negative. When considering VLP/NS1 OD ratios (R), the obtained results also indicated that this parameter was not predictive enough in this instance to differentiate between infected and vaccinated horses. Although the majority of the cases behave as expected, i.e., most infected horses had R< 4, and, conversely, in most of the vaccinated ones these R values were higher than 4, still some infected and vaccinated horses did not follow this trend, but in fact did the opposite; i.e., there were relevant percentages of infected horses displaying R>4 and of vaccinated horses displaying R<4. By groups, for the naturally infected horses differences were not relevant with regard to the country of origin, but in horses vaccinated in the field, differences arose between different types of vaccines and vaccination schedules. Overall, two types of vaccines were analysed: inactivated whole virus vaccine and live recombinant virus vaccine. Also, different administration schedules were taken into account: a single administration schedule (groups B2, B3, and B6) or a recall vaccination one year after the first administration (groups B4 and B5), and, in this latter case, there were two different schedules: one using the same formulation as in primovaccination (group B4) and the other using a different vaccine (inactivated) from the one provided first (recombinant) (group B5). Overall, single-vaccinated animals had higher percentages of sera behaving as “infected” (i.e., R<4) than animals provided with a recall vaccination. Amongst single-vaccinated animals, two behaviours can be outlined: on one hand, horses receiving inactivated vaccines (B2 and B6), associated with moderately high percentages of sera with R<4 (30 to 50%), and, on the other hand, horses receiving a single administration of recombinant vaccine (B3), associated with 100% of sera with R<4. In this latter case, behaving exactly as it would have been expected not for vaccinated but for infected animals, the result is apparently related not to the presence of NS1 antibodies, which were apparently absent, but to an inconsistently low antibody response to structural WNV proteins, likely due to an underdeveloped immunity elicited by the single vaccination provided. The results observed changed completely in the group provided with a recall vaccine of the same type as the first (B4), with a stronger antibody response to the structural proteins. Even stronger immunity to structural proteins without rising of NS1 antibodies, was observed in group B5, receiving a recall vaccination with inactivated vaccine while it was first vaccinated with the recombinant one. Apparently, this schedule performed quite well as far as the DIVA results are concerned, since all samples from this group had high R values, above 4, as expected, at least theoretically, from vaccinated animals. However, this result would require further analyses with larger populations since it is supported by a small number of samples.

Overall, these findings hardly support the use of NS1 as a marker for DIVA methods, at least in the conditions prevailing in the field where the samples under study came from and with the types of vaccines and vaccine schedules included. In this study the samples are from four Western-Mediterranean countries having areas affected by WNV foci since at least 8 years, and in some instances more time. In those areas, horses are vaccinated by the owners based on risk perception, that is, when the virus is circulating nearby. This circumstance may affect the results observed in the study since, in some cases, vaccination of infected, asymptomatic equines may occur under these conditions, which may explain the percentage of vaccinates (37.4%) behaving serologically as infected, i.e., NS1-antibody positive or R< 4. However, the existence of a small percentage of infected equines (20%) behaving as vaccinates (i.e., NS1-antibody negative or R> 4) is more difficult to explain. In the Western-Mediterranean areas affected by WNV circulation, there are other flaviviruses currently circulating, such as Usutu virus, Bagaza virus, Meaban virus, or tick-borne encephalitis virus, which can cross-react with WNV in serological tests [14]. Since NS1 antibody response appears to be, to some extent, flavivirus specific, as judged by the results published by Cleton and cols [7], it may occur that horses infected with one of these non-WNV flaviviruses could raise antibodies cross-reacting with structural antigens, but not with NS1, thus providing a satisfactory explanation to the findings revealed in this study.

## 5. Conclusions

In animal health in general and in West Nile virus disease in particular, the differentiation between infected and vaccinated animals through the analysis of the antibody response against nonstructural proteins seems a good strategy, worth pursuing. This study showed that despite correct differentiation between WNV-infected and vaccinated horses was possible in experimental (controlled) conditions using NS1 antigen as infection marker, this was not possible using field sera from countries from the Western-Mediterranean region, where both infection and vaccination are widespread, and non-WNV cross-reacting flaviviruses circulate in the same areas as WNV. Both circumstances could potentially affect the performance of NS1-based DIVA methods. Nevertheless, new DIVA strategies are worth exploring, which may be based on different nonstructural protein antigens or vaccines marked with a differential antigen.

## Figures and Tables

**Table 1:** Details of the horse serum samples used in this study.

***WNV strain or vaccine***	***Sample group number***	***Infection mode***	***Nº of serum samples***	***Nº of animals***	***dpi/dpv***	***Source/origin***	***Confirmatory test***
			**Experimental**				
**Israel '98 (L1)**	A1	Experimental	8	1	0-58	France	IgG,IgM,VNT
**Austria '08 (L2)**	A1	Experimental	8	1	0-58	France	IgG,IgM,VNT
**Equip ** **W** **N** **V** ^**a**^	B1	Vaccine (experimental)	50	5	0-115	Spain	
			**Field**				
**Unknown**	A2	Natural	22	22		France	IgG, IgM
＇＇	A3	Natural	17	17		Italy	IgM
＇＇	A4	Natural	24	24		Morocco	IgM,IgG
＇＇	A5	Natural	52	52		Spain	IgM, VNT
**Equip ** **W** **N** **V** ^**a**^	B2	Vaccine	26	26		Spain	
**Proteq WNV **1**x**^**b**^	B3	Vaccine	30	30		Spain	
**Proteq WNV**2**x**^**b**^	B4	Vaccine	76	76		Spain	
**WNVVa** **c** ^**c**^	B5	Vaccine	13	13		Morocco	
**Recombite** **k** ^**d**^ ** + WNVac**	B6	Vaccine	19	19		Morocco	
**None**	C	Field (no exposure to WNV)	96	96		Spain	

a. Duvaxyne WNV (called now Equip WNV) commercially available by Zoetis. Inactivated WNV, strain VM-2 injectable. Administration route 2 doses IM/annual recalled.

b. Proteq WNV commercially available by Merial. Recombinant virus canary pox containing PrM-E WNV proteins. Administration route 2 doses IM/annual recalled.

c. WNV Vac commercially available by Biopharma. Inactivated WNV, strain Maroc 96-107 injectable. Administration route 2 doses IM/annual recalled.

d. Recombitek equine WNV. The same vaccine as Proteq WNV (b) but lyophilized presentation.

**Table 2:** Results of serological examinations in field samples from WNV infected and vaccinated horses.

*Condition*	*Subgroup*	*Nº of sera*	*Anti-NS1*		*Anti VLP*		*Ratio*	
	* (by origin/ vaccine type)*				*(mean OD)*		*OD(VLP)/OD(NS1)*	
			*OD *	*Posit* ^*∗*^ * *	*OD *	*Posit* ^*∗*^ * *	*R<4 *	*R>4 *
			* (mean+s.d.)*	* n (%)*	* (mean+s.d.)*	*n (%)*	* n (%)*	* n (%)*
*Field-acquired infection*	A2 (France)	22	0.75±0.46	14 (63.6)	1.29±0.89	22 (100)	20 (90.9)	2 (9.1)
	A3 (Italy)	17	0.45±0.28	6 (35.3)	1.42±0.59	17 (100)	10 (58.8)	7 (41.2)
	A4 (Morocco)	24	0.69±0.29	17 (70.1)	1.52±0.66	24 (100)	21 (87.5)	3 (12.5)
	A5 (Spain)	52	0.97±0.62	40 (76.9)	2.79±1.73	52 (100)	41 (78.8)	11 (20.0)
	**TOTAL (A2-A5)**	**115**	**0.72±0.41**	**77 (66.9)**	**1.75±0.97**	**115 (100)**	**92 (80.0)**	**23 (20.0)**

*Field vaccination*	B2 (Equip)	26	0.29±0.29	4 (15.4)	1.16±0.52	25 (96.1)	13 (50.0)	13 (50.0)
	B3 (Proteq x1)	30	0.42±0.2	9 (30.0)	0.72±0.27	27 (90)	30 (100)	0 (0)
	B4 (Proteq x2)	75	0.23±0.28	4 (5.3)	1.75±0.57	75 (100)	14 (18.6)	61 (81.3)
	B5 (Recombitek +WNVvac)	19	0.13±0.10	0 (0)	2.08±0.33	19 (100)	0 (0)	19 (100)
	B6 (WNVvacB6)	13	0.43±0.39	5 (38.4)	2.50±0.26	13 (100)	4 (30.7)	9 (69.2)
	**TOTAL (B2+B3+B4+B5+B6)**	**163**	**0.31±0.27**	**22 (13.4)**	**1.24±0.47**	**162 (98.7)**	**61 (37.4)**	**102 (62.6)**

e. (*∗*) Positives (Indirect ELISA): values above 0.5 OD units.

## Data Availability

The data used to support the findings of this study are available from the corresponding author upon request.
